# Anti-inflammatory and Antioxidant Activity of Neem and Kirata-Induced Silver Nanoparticles Against Oral Biofilm: An In Vitro Study

**DOI:** 10.7759/cureus.67708

**Published:** 2024-08-25

**Authors:** Rebekah Raju, Arya S Prasad, Rajesh Kumar S

**Affiliations:** 1 Department of Orthodontics and Dentofacial Orthopaedics, Saveetha Dental College and Hospital, Saveetha Institute of Medical and Technical Sciences, Saveetha University, Chennai, IND; 2 Department of Pharmacology, Saveetha Dental College and Hospital, Saveetha Institute of Medical and Technical Sciences, Saveetha University, Chennai, IND

**Keywords:** biofilm, antioxidant property, anti-inflammatory property, neem and kirata, nanoparticles

## Abstract

Introduction

Silver nanoparticles have been the most commonly used nanoparticles which could be integrated with plant extracts. The mutually beneficial interaction between neutral plant extracts and nanoparticles reduced the chemical toxicity while promoting synthesis. Azadirachta indica, widely known as the neem plant, has diverse medicinal compounds encompassing antibacterial, antiviral, antiprotozoal, insecticidal, antifungal, and antioxidant properties. Swertia chirata, known as Chirayata in India, stands out for its dual roles as a laxative and appetiser with pronounced antimicrobial and anti-inflammatory qualities. Hence, this study aimed to evaluate the anti-inflammatory and antioxidant properties of silver nanoparticles synthesized using Neem and Kirata extract.

Materials and methods

The plant extracts of Neem (Azadirachta indica) and Kirata (Swertia chirata) were obtained in powder form. It was later formulated into an extract and stored in a refrigerator at 4 degrees Celsius. The formulated extract of Neem and Kirata was then incorporated with silver nitrate to form a modified silver nanoparticle using a green synthesis approach. The anti-inflammatory activity of Neem and Kirata extract was tested using Bovine Serum Assay and Egg Albumin Assay. The antioxidant activity of the new herbal-formulated Ag nanoparticles was determined by the DPPH ((2,2-diphenyl-1-picrylhydrazyl) assay.

Results

Based on the anti-inflammatory assays, the Neem and Kirata-induced nanoparticles showed increasing levels of inhibition, while the standard showed slightly higher inhibition at 10, 20 and 30 µL. At 40 µL and 50 µL, both Kirata and Neem (Ag) and the standard showed high levels of inhibition, nearing 75% and above, with the standard consistently showing a marginally higher inhibition percentage. Based on the DPPH assay, the Neem Kirata-induced Ag nanoparticle showed a comparable or slightly higher inhibition percentage compared to the standard.

Conclusion

The study underscores the potential of Neem and Kirata herbal-based silver nanoparticles as effective anti-inflammatory and antioxidant agents. Future research directions should focus on refining nanoparticle synthesis, investigating mechanisms of action, and exploring additional therapeutic applications in the biomedical and pharmaceutical sectors.

## Introduction

Nanoscience refers to the investigation of applied science at the atomic or molecular level [1]. However, nanotechnology's wide-ranging influence has been extended to many aspects of modern life, including the medical sector. Nano dentistry, akin to nanomedicine, harnesses nanomaterials and biotechnologies to advance oral health [2]. Nanoparticles could be synthesized using chemical, physical methods or green synthesis methods. The benefits of green synthesis include its non-toxic, environmental-friendly, economical and sustainable properties. The drawbacks involve issues in the extraction of raw materials, reaction time and quality of final products. Silver nanoparticles (AgNPs) have been the most commonly used nanoparticles which could be integrated with plant extracts. Despite the limitations, green synthesis has been proven to be an eco-friendly alternative in nanoparticle synthesis [3]. Silver nanoparticles (AgNPs), among noble metal nanoparticles, have been widely used for their multifaceted applications in medicine, dentistry, pharmaceuticals, tissue imaging, tumor detection, biolabeling, and more. Extensive literature highlighted the AgNP's antimicrobial properties across antibacterial, antifungal, antiviral, and antiparasitic realms [4,5]. The mutually beneficial interaction between neutral plant extracts and nanoparticles reduced chemical toxicity while promoting synthesis. Plant-derived compounds, renowned for their anti-inflammatory and antioxidant attributes, have been commonly utilized [4]. Azadirachta indica, widely known as the neem plant, has diverse medicinal compounds, including azadirachtin, nimbinene, nimbin, nimbandial, diacetyl nimbinase, and salanin, belonging to the terpenoid group, alongside phenolic compounds like quercetin, rutin, and gallic acid. These compounds demonstrate a spectrum of effects encompassing antibacterial, antiviral, antiprotozoal, insecticidal, antifungal, and antioxidant properties [5]. Swertia chirata, known as Chirayata in India, stands out for its dual roles as a laxative and appetizer with pronounced antimicrobial and anti-inflammatory qualities [6]. Neem and neem-related products have been used to target pathogens resistant to first-line antibiotics, oral health-related bacterial species that create hard-to-remove biofilms, and against other fungal and viral infections. A previous study was performed in order to characterize the Neem and Kirata-induced nanoparticle and evaluate its antimicrobial and cytotoxic properties [7]. But further research was needed in order to confirm its beneficial properties and prove its efficacy as an apparent biological compound against the oral microbiofilm. Oral biofilm is a predisposing factor for various oral infections such as gingivitis, dental caries, periapical periodontitis, periodontitis, and peri-implantitis. Hence the aim of the study was to assess the anti-inflammatory and antioxidant properties of Neem and Kirata-induced silver nanoparticles thereby resisting the formation of oral biofilm and preventing their deleterious effects.

## Materials and methods

Ethical approval

The study was approved by the institutional review board of the Saveetha Institute of Medical and Technical Sciences (IEC number: SRB/SDC/ORTHO-2101/22/061).

Experimental setup

This experiment was conducted to synthesize nanoparticles using Neem and Kirata plant extracts and to evaluate the anti-inflammatory and antioxidant activities of the green synthesized nanoparticle. The study was performed at the nanoparticle research laboratory at Saveetha Dental College, Chennai. The plant extracts of Neem (Azadirachta indica) and Kirata (Swertia chirata) were obtained in powder form from a recognized herbal manufacturing unit in South India.

Formulation of the New Extract

**Figure 1:**
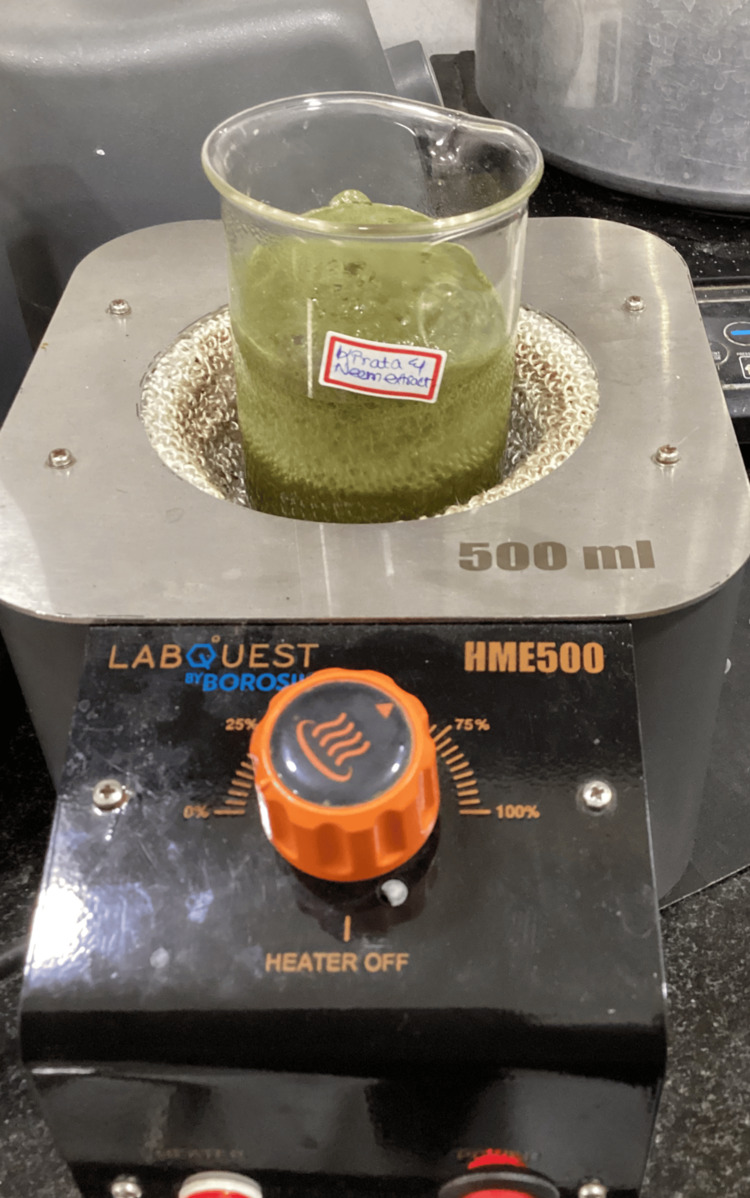
Neem and Kirata boiling extract

Neem and Kirata plants were procured in powdered form in equal quantities. Two grams of Neem and Kirata powders were separately acquired, and measured in a standard weighing balance. The powdered extracts were dissolved in conical flasks by incorporating 100 mL of distilled water. The amalgam was then subjected to heating on a mantle until it attained a boiling temperature of 60 degrees Celsius (Figure 1). Post-boiling, the extract was filtered using filter paper. The resulting purified extract was then collected and stored in a refrigerator at 4 degrees Celsius.

Synthesis of Modified Silver Nanoparticle

**Figure 2:**
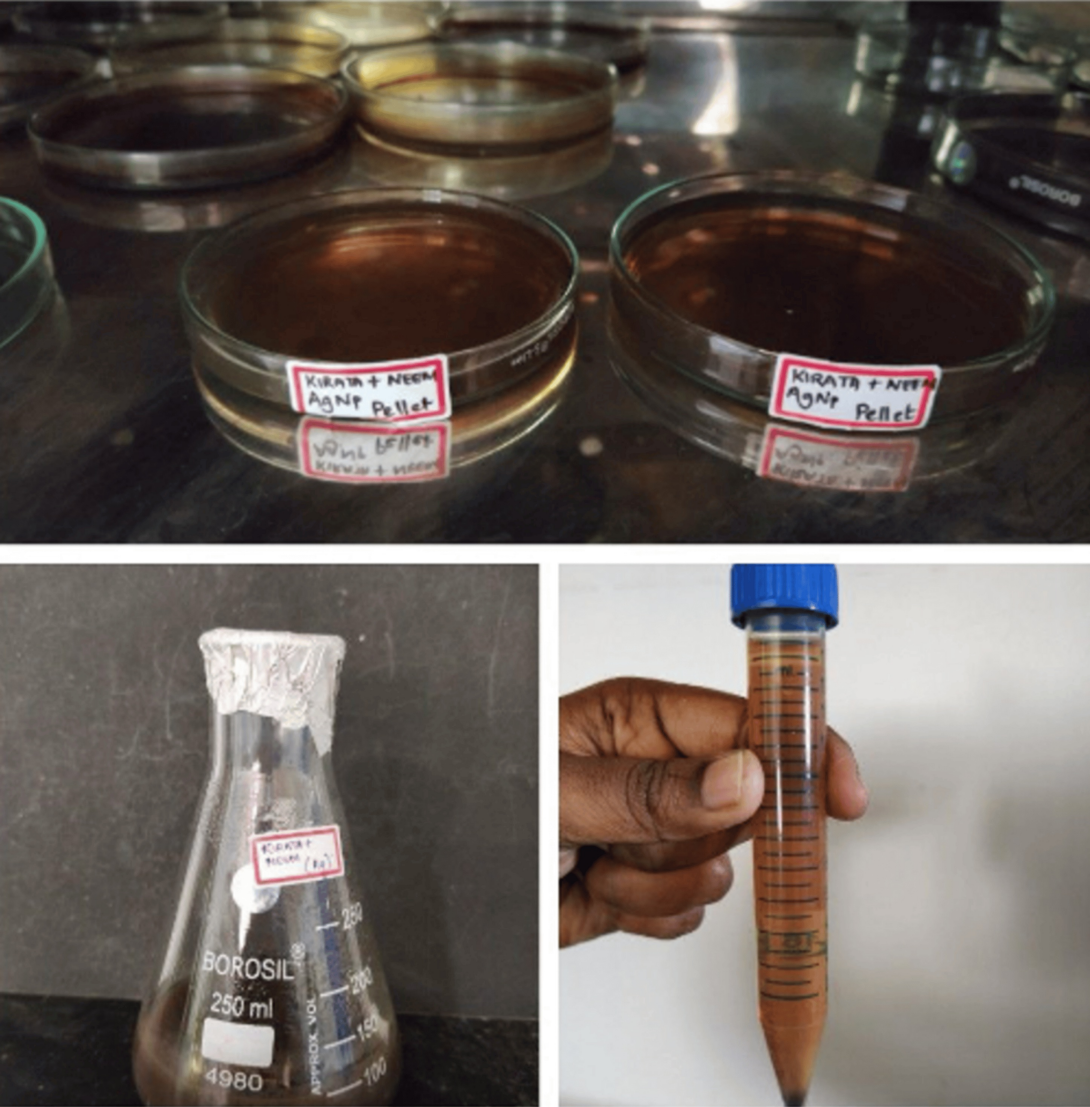
Neem and Kirata Ag nanoparticle synthesis

The newly formulated extract of Neem and Kirata was then incorporated with silver nitrate to form a modified silver nanoparticle. It underwent a process involving treatment with 0.016 g of silver nitrate and 90 ml of distilled water. Following this treatment, the extract was subjected to agitation on an orbital shaker at 120 rpm overnight. The synthesis of nanoparticles was then observed regularly, with measurements taken at hourly intervals using a double-beam UV spectrometer covering wavelengths from 250 to 650 nm. After synthesizing the nanoparticle solution, it was subjected to centrifugation at 8000 rpm for 10 min. Upon completion of this step, the supernatant and pellet were separated, with the latter being retained for storage (Figure 2).

Activity against inflammation

Bovine Serum Assay

The anti-inflammatory activity of Neem and Kirata extract was tested using a similar experiment previously conducted by Muzushima and Kabayashi with certain specific changes. Neem and Kirata extract of 0.05 mL of different concentrations (10 µL, 20 µL, 30 µL, 40 µL, 50 µL) were mixed with 1% aqueous solution of 0.45 mL bovine serum albumin. The solutions were brought to a pH of 6.3 using 1N hydrochloric acid. They were later incubated for 20 min at room temperature and then heated in a water bath for 30 min at 55°C. After cooling, the absorbance of each sample was measured spectrophotometrically at 660 nm.

Egg Albumin Assay

The egg albumin assay was also conducted to assess the anti-inflammatory activity. To cause denaturation of the egg albumin, the samples were heated in a water bath for 20 min at 70°C after being incubated for 10 min at 37°C. Diclofenac sodium served as a standard anti-inflammatory drug for comparison. DMSO was used as a control. The mixture was cooled before the absorbance at 660 nm was measured.

The protein denaturation was determined by the formula:

% inhibition = [(Absorbance of control - Absorbance of sample)/Absorbance of control] ×100

Activity against oxidation

The antioxidant activity of the new herbal-formulated Ag nanoparticles was determined by DPPH ((2,2-diphenyl-1-picrylhydrazyl) assay. Increasing concentrations (10-50 μg/ml) of Neem and Kirata-induced silver nanoparticle was mixed with 1 ml of 0.1 mM DPPH in methanol and Tris HCl buffer (50 mM at pH 7.4) of the quantity of 450 μL and placed for incubation for a period of 30 min. The decrease of DPPH free radicals was determined based on the absorbance noted at 517 nm. Butylated hydroxytoluene (BHT), a known antioxidant, was used as a positive control to validate the assay and compare the antioxidant activity of the silver nanoparticles.

The inhibition percentage was calculated by the following formula:

% inhibition = [(Absorbance of control - Absorbance of test sample)/Absorbance of control] × 100

## Results

Anti-inflammatory activity

**Figure 3:**
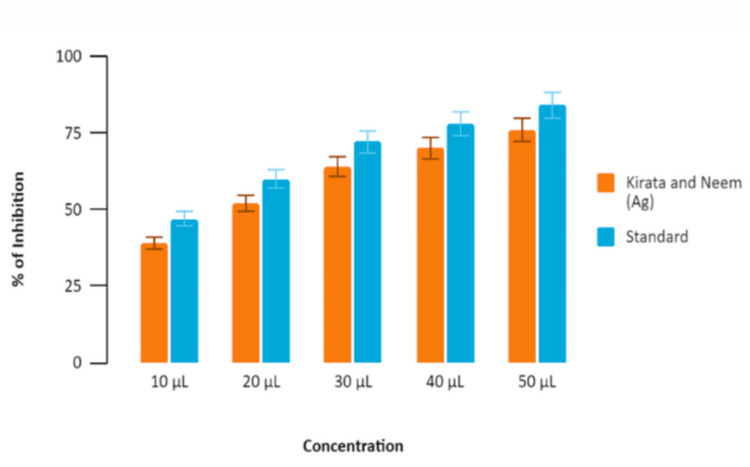
Bovine Serum Assay (BSA assay) Percentage of inhibition based on the concentration of nanoparticle

**Figure 4:**
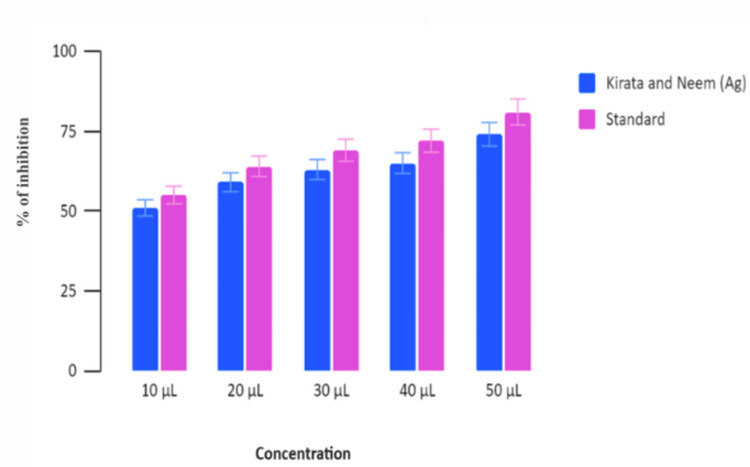
Egg Albumin assay (EA assay) Percentage of inhibition based on nanoparticle concentration

Kirata and Neem-induced Ag nanoparticles were compared to a standard across varying concentrations (10 µL, 20 µL, 30 µL, 40 µL, and 50 µL). The percentage of inhibition increased for both the nanoparticle and the standard as the concentration increased from 10 µL to 50 µL. At 10, 20 and 30 µL, the Neem and Kirata-induced nanoparticles showed increasing levels of inhibition, while the standard showed slightly higher inhibition. At 40 µL and 50 µL, both Kirata and Neem (Ag) and the standard show high levels of inhibition, nearing 75% and above, with the standard consistently showing a marginally higher inhibition percentage. The percentage of inhibition observed for Neem and Kirata-induced Ag nanoparticles compared to a standard across varying concentrations (10 µL, 20 µL, 30 µL, 40 µL, and 50 µL) has been graphically illustrated in Figure 3. Similar results were obtained for the Egg Albumin Assay, where both standard and Neem and Kirata Ag nanoparticles showed higher concentrations, with Standard treatment showing consistently higher effectiveness as shown in Figure 4.

Antioxidant activity

**Figure 5:**
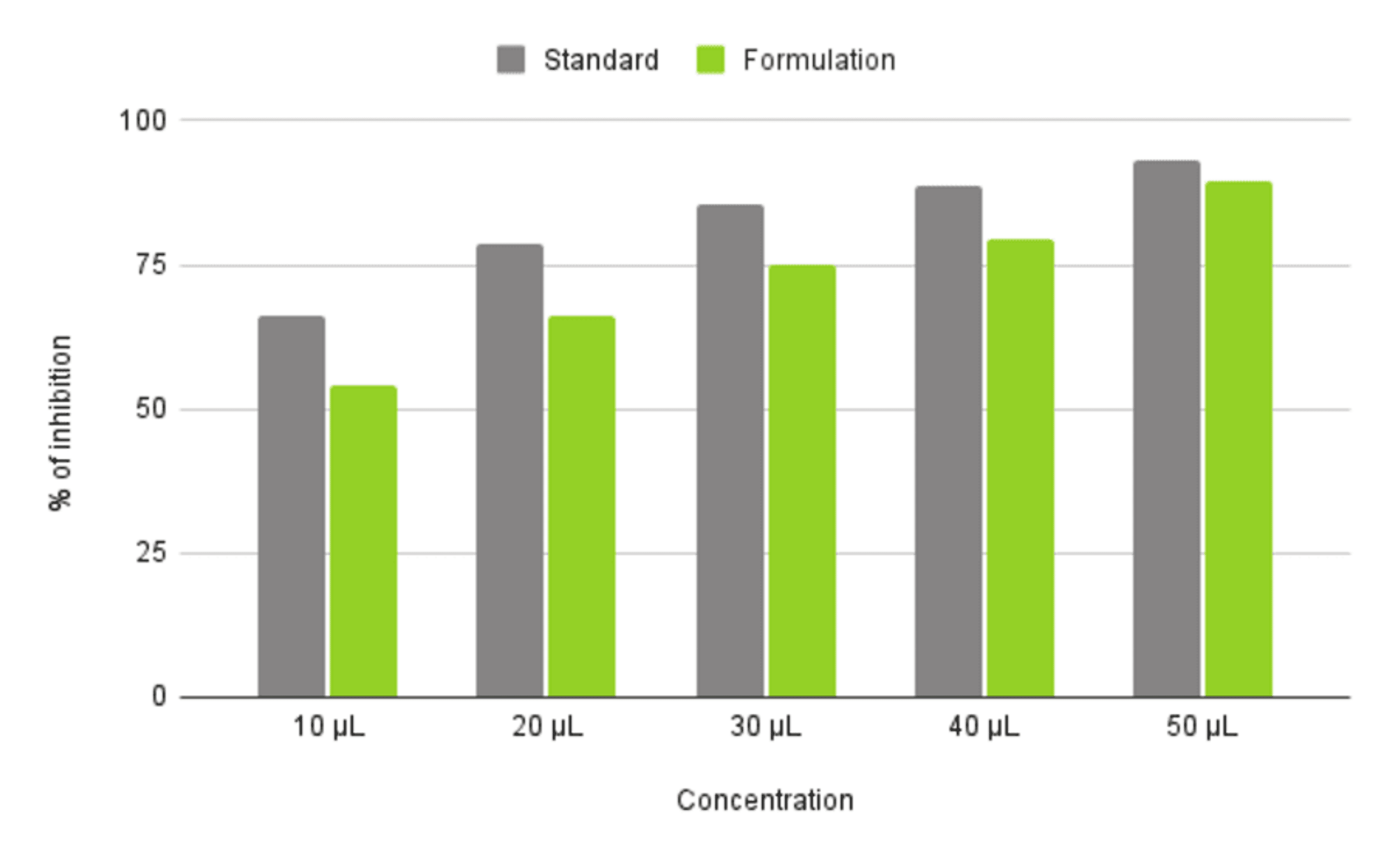
Antioxidant activity Percentage of inhibition based on nanoparticle concentration.

Based on the DPPH assay, the reduction in DPPH free radicals was determined using BHT (butylated hydroxytoluene) as a control and the absorbance level at 517 nm. Figure 5 displays the percentage of inhibition at various doses (10 µL-50 µL). At lower concentrations (10 µL and 20 µL), the standard treatment showed higher inhibition than the modified nanoparticle formulation. At higher concentrations (30 µL, 40 µL, and 50 µL), the Neem Kirata-induced Ag nanoparticle showed a comparable or slightly higher inhibition percentage compared to the standard.

## Discussion

This in vitro study examined the attributes of Neem and Kirata herbal-based silver nanoparticles concerning their impact on inflammation and oxidation. Based on the in vitro activity, the BSA assay resulted in the highest inhibition at 50 µL concentration, depicting higher anti-inflammatory activity. On the EA assay, the anti-inflammatory activity was found to be significant with an increase in the activity with increasing concentrations. Though the increase in the concentration was lesser than the standard, the newly formulated nanoparticle showed promising results almost comparable to the standard. Based on the DPPH assay, the newly formulated nanoparticle showed a high percentage of inhibition with about 85% in 50 µL concentration, superior to the standard.

There have been previous studies that have quoted the increased anti-inflammatory and antioxidant properties of Neem. Girish and Shankara Bhat stated that Neem was used as a traditional medicine and is beneficial as it is biocompatible in nature [8]. While analysing the anti-inflammatory property, Khetarpal et al. stated that Neem along with aloe vera and curcumin possessed significant anti-inflammatory activity [9]. Subapriya and Nagini described various pharmacological activities of Neem against inflammation, oxidation, ulcers, carcinogens, and immunomodulatory as well as antibacterial properties [5]. The nanoparticles created in the synthesis process effectively hindered protein denaturation, showcasing a significant inhibition rate of 75.52% at a concentration of 500 μg ml, whereas standard drug Aspirin exhibited 65.03% protein denaturation [10]. Kaur et al. stated that during an in vitro exposure, Nimbidin, a component of Neem leaf, inhibited nitric oxide and the production of prostaglandins in lipopolysaccharide-stimulated macrophages [11]. The anti-oxidant property was also found to be superior to other plant extracts. Sithisarn et al. suggested that there was high antioxidant activity seen in the leaf, flower, bark and stem of the Neem plant [12]. In another study by Dhakal et al., it was observed that the methanolic extracts of Neem possessed high anti-oxidant activity [13].

On the other hand, Swertia chirata was also one of the most widely used plant herbs for various medical conditions. Hossain et al. mentioned that ethanolic extracts consisting of Swertia chirata showed high thrombolytic and anti-inflammatory activity in in vitro conditions [14]. Swertia chirata consisted of various active components such as swerchirin, swertianin, and swertanone which increased the anti-inflammatory property [15]. Mangiferin, one of the components, had high activity against inflammation and it caused good changes in the joints of arthritic mice [15,16]. In a comparative study by Ghimeray et al., Swertia chirata showed high activity against oxidation [17]. The ethanolic extract of chirata also possesses an antioxidant effect, which aids Swertia chirata in curing liver diseases [18]. Mohammad et al. conducted a study indicating that various fractions of the methanolic extract from Swertia chirata exhibited antioxidant, antimicrobial, and cytotoxic effects on brine shrimp, along with anti-leishmanial activities [19]. Additionally, silver nanoparticles synthesized from herbal formulations of Andrographis paniculata and Phyllanthus niruri exhibited promising activity against oral microorganisms [20]. In a study by Johnson et al., it was observed that the presence of surfactin-loaded nanoparticles led to a decline in the production of transcription factors, cytokines, and reactive oxygen species (ROS) [21]. Abdelbaky et al. suggested in their research that biosynthesized ZnO nanoparticles could serve as promising resources for antioxidants, antibacterial properties, and anti-inflammatory applications in the biomedical and pharmaceutical sectors [22].

Compared with other studies evaluating the properties of Neem and Kirata individually, this study combined the phytochemical properties of both Neem and Kirata which improved the overall characteristics of the newly formed nanoparticle. There has been no other study that has depicted the combined effects of these plant extracts against oxidation and inflammatory properties.

Limitations and future scope

The present study had certain limitations, including burst drug release, low stability of the formulated nanoparticles and cytotoxic effects of the plant extracts in varying concentrations. Being a qualitative in-vitro study, the sample size calculation was beyond the scope of this study. Despite their limitations, the amalgamation of Neem and Kirata is anticipated to produce favourable outcomes, including heightened anti-inflammatory and antioxidant effects. As a result, silver nanoparticles derived from Neem and Kirata have the potential to offer diverse beneficial effects, particularly in countering oral pathogens. Hence future approaches could develop more efficient nanoparticles from a large type of plants while optimizing the process of elaboration and valorization with a coherent protocol for use in various biomedical applications.

## Conclusions

The study indicated that silver nanoparticles derived from Neem and Kirata possessed anti-inflammatory and antioxidant qualities, displaying significant inhibitory effects with few associated adverse effects. The newly formulated nanoparticle has the potential to have anti-inflammatory properties thereby reducing the inflammatory mediators, gingival inflammation and pathological adhesion. The antioxidant properties help in the neutralization of free radicals thereby reducing oxidative stress and stabilization of the oral environment. Hence, this research lays a substantial foundation for understanding the characteristics and origins of plant-modified silver nanoparticles and their effect on the oral biofilm.

## References

[1] Inamuddin Inamuddin, Boddula R, Asiri AM (2024). Actuators: Fundamentals, Principles, Materials and Applications. https://onlinelibrary.wiley.com/doi/book/10.1002/9781119662693.

[2] Ozak ST, Ozkan P (2013). Nanotechnology and dentistry. Eur J Dent.

[3] Ying S, Guan Z, Ofoegbu PC, Clubb P, Rico C, He F, Hong J (2022). Green synthesis of nanoparticles: current developments and limitations. Environ Technol Innov.

[4] Mérillon JM, Riviere C (2018). Natural Antimicrobial Agents. Sustainable Development and Biodiversity. Natural Antimicrobial Agents. Sustainable Development and Biodiversity.

[5] Subapriya R, Nagini S (2005). Medicinal properties of neem leaves: a review. Curr Med Chem Anticancer Agents.

[6] Kumar V, Van Staden J (2015). A review of Swertia chirayita (Gentianaceae) as a traditional medicinal plant. Front Pharmacol.

[7] Rebekah R, Saravana Dinesh SP, Nivethigaa B, Rajeshkumar S (2022). In vitro antimicrobial and cytotoxic activity of Neem and Kirata herbal formulation mediated Silver nanoparticles. Bioinformation.

[8] Girish K, Shankara Bhat S (2008). Neem - A green treasure. Electronic J Biol.

[9] Khetarpal S, Bansal A, Kukreja N (2014). Comparison of anti-bacterial and anti-inflammatory properties of neem, curcumin and aloe vera in conjunction with chlorhexidine as an intracanal medicament - an in-vivo study. Dental J Adv Studies.

[10] Patil PA, Dalvi S, Dhaygude V, Shete SD (2022). Formulation of silver nanoparticle of Cassia angustifolia by using green synthesis method and screening for in-vitro anti-inflammatory activity. Indo Global J Pharm Sci.

[11] Kaur G, Sarwar Alam M, Athar M (2004). Nimbidin suppresses functions of macrophages and neutrophils: relevance to its antiinflammatory mechanisms. Phytother Res.

[12] Sithisarn P, Supabphol R, Gritsanapan W (2005). Antioxidant activity of Siamese neem tree (VP1209). J Ethnopharmacol.

[13] Dhakal S, Aryal P, Aryal S, Bashyal D, Khadka D (2016). Phytochemical and antioxidant studies of methanol and chloroform extract from leaves of Azadirachta indica A. Juss. in tropical region of Nepal. J Pharmacogn Phytother.

[14] Hossain MS, Chowdhury ME, Das S, Chowdhury IU (2012). In-vitro thrombolytic and anti-inflammatory activity of Swertia chirata ethanolic extract. J Pharmacogn Phytochem.

[15] Rathore B, Ali Mahdi A, Nath Paul B, Narayan Saxena P, Kumar Das S (2007). Indian herbal medicines: possible potent therapeutic agents for rheumatoid arthritis. J Clin Biochem Nutr.

[16] Kumar IV, Paul BN, Asthana R, Saxena A, Mehrotra S, Rajan G (2003). Swertia chirayita mediated modulation of interleukin-1beta, interleukin-6, interleukin-10, interferon-gamma, and tumor necrosis factor-alpha in arthritic mice. Immunopharmacol Immunotoxicol.

[17] Ghimeray AK, Jin C, Ghimire BK, Cho DH (2009). Antioxidant activity and quantitative estimation of azadirachtin and nimbin in Azadirachta indica A. Juss grown in foothills of Nepal. Afr J Biotechnol.

[18] Chen Y, Huang B, He J, Han L, Zhan Y, Wang Y (2011). In vitro and in vivo antioxidant effects of the ethanolic extract of Swertia chirayita. J Ethnopharmacol.

[19] Akhter SMH, Mahmood Z, Ahmad S, Mohammad F (2018). Plant-mediated green synthesis of zinc oxide nanoparticles using Swertia chirayita leaf Extract, characterization and Its antibacterial efficacy against some common pathogenic bacteria. BioNanoSci.

[20] Selvapriya S, Monika K, Rajeshkumar S (2020). Antioxidant activity of silver nanoparticles synthesis using Cinnamomum verum and Phyllanthus emblica formulation. Int J Res Pharm Sci.

[21] Johnson A, Kong F, Miao S, Thomas S, Ansar S, Kong ZL (2021). In-vitro antibacterial and anti-inflammatory effects of surfactin-loaded nanoparticles for periodontitis treatment. Nanomaterials (Basel).

[22] Abdelbaky AS, Abd El-Mageed TA, Babalghith AO, Selim S, Mohamed AM (2022). Green synthesis and characterization of ZnO nanoparticles using Pelargonium odoratissimum (L.) aqueous leaf extract and their antioxidant, antibacterial and anti-inflammatory activities. Antioxidants (Basel).

